# Beyond the “Master” Role in Allergy: Insights into Intestinal Mast Cell Plasticity and Gastrointestinal Diseases

**DOI:** 10.3390/biomedicines13020320

**Published:** 2025-01-29

**Authors:** Rosa Molfetta, Alessia Carnevale, Caterina Marangio, Erisa Putro, Rossella Paolini

**Affiliations:** Department of Molecular Medicine, Laboratory Affiliated to Istituto Pasteur Italia-Fondazione Cenci Bolognetti, Sapienza University of Rome, 00161 Rome, Italy; rosa.molfetta@uniroma1.it (R.M.); a.carnevale@uniroma1.it (A.C.); caterina.marangio@uniroma1.it (C.M.); erisa.putro@uniroma1.it (E.P.)

**Keywords:** intestinal mast cells, homeostasis, gut inflammation

## Abstract

Mast cells (MCs) are essential components of the immune system that enter the circulation as immature bone marrow progenitors and differentiate in peripheral organs under the influence of microenvironment factors. As tissue-resident secretory immune cells, MCs rapidly detect the presence of bacteria and parasites because they harbor many surface receptors, which enable their activation via a multitude of stimuli. MC activation has been traditionally linked to IgE-mediated allergic reactions, but MCs play a pivotal role in different physiological and pathological processes. In gut, MCs are essential for the maintenance of gastrointestinal (GI) barrier function, and their interactions with neurons, immune cells, and epithelial cells have been related to various GI disorders. This review recapitulates intestinal MC roles in diseases with a main focus on inflammatory bowel disease (IBD) and irritable bowel syndrome (IBS). Emerging therapies targeting MCs and their mediators in clinical practices will also be discussed.

## 1. Introduction

It has been known for decades that mast cells (MCs) are key players in immunoglobulin E (IgE)-dependent allergic disorders, including asthma and systemic anaphylaxis [1]. However, their participation in inflammatory bowel disease (IBD) and irritable bowel syndrome (IBS) is less appreciated. One reason is that a clear link between MC-restricted genes and IBD has not been revealed by genome-wide association studies [2]. Another reason for the lack of appreciation is that cationic stains (e.g., toluidine blue, safranin, and methylene blue) routinely performed on biopsies of connective tissues to quantitate MCs are not particularly effective for the identification of these cells in the gastrointestinal tract [3].

Despite these difficulties, an essential role for mouse and human MCs and their proteases in promoting barrier dysfunctions has been documented either in acute experimental colitis and in gastrointestinal disorders including IBD and IBS [4,5,6]. Moreover, the use of different antibody combinations able to detect proteins restricted to human MCs (e.g., the granule proteases tryptase and chymase and the surface receptor c-kit) has finally permitted the characterization of the distribution and phenotype of MCs in gut biopsies of patients with IBD [7].

Thus, it is now becoming clear that MCs play a major role in the regulation of intestinal mucosal permeability, in the initiation and maintenance of neuro-immune interaction and inflammatory response in the gut, as well as in tissue remodeling after resolution of the acute inflammatory phase.

In this review, we first describe the biological properties of MCs in the gastrointestinal tract and their essential role in preserving homeostatic conditions. We will then discuss the contribution of MCs and their selective mediators in the inflammation that occurs in the gut of patients with IBD and IBS. The review also highlights the potential clinical applications of recent findings.

## 2. Phenotypic and Functional Heterogeneity of Intestinal MCs

MCs are myeloid cells that originate from poorly granulated CD34^+^/c-Kit^+^ bone marrow progenitors that enter the circulation and complete their maturation into peripheral tissues under the influence of the local microenvironment [8,9]. Notably, in the mouse, the greatest number of MC-committed progenitors (MCp) are found in the gut compared to other peripheral tissues [10].

The homing of murine MCp in the intestine is driven by the expression of the α_4_β_7_ integrin and the chemokine receptor CXCR2 on the surface of the progenitors and the expression of Madcam1 and VCAM1 on the intestinal endothelium [11,12]. However, the most important signaling pathway that controls the retention and viability of MCp in the GI tract is that between the tyrosine kinase receptor c-Kit (CD117) on MCs and c-Kit ligand (stem cell factor, SCF) on the plasma membranes of fibroblasts, endothelial cells, and other stromal cells [13,14]. Additionally, several other cytokines [e.g., interleukin (IL)-3, IL-4, IL-6, IL-9, IL-10, IL-33, nerve growth factor, and transforming growth factor-β] are needed for the final stages of differentiation and for the development of phenotypically distinct populations of mouse and human MCs [15,16,17,18,19,20]. 

Mature MCs express an extensive array of receptors that allow them to recognize invading pathogens and respond to different stimuli coming from the microenvironment [21,22]. Triggering of these receptors leads to exocytosis of granule-stored mediators or the generation of newly biologically active lipid mediators (e.g., leukotrienes, prostaglandins, thromboxane, and platelet-activating factor) [23,24,25]. Moreover, activated MCs can release numerous cytokines and chemokines orchestrating the delayed phase of inflammation that occurs several hours after their initial stimulation.

The high-affinity receptor for IgE (FcεRI) binds monomeric IgE and is the main activating receptor constitutively expressed on the surface of MCs. Multivalent antigen crosslinking of the receptor-bound IgE leads to activation of signaling pathways that are the primary cause of MC-dependent hypersensitivity reactions in vivo, including systemic anaphylaxis [26,27].

Mouse and human MCs can also be activated in many non-IgE-dependent manners [25]. For instance, MCs can be induced to degranulate by thrombin via protease-activated receptor-1 (Par-1) [28,29], by IgG complexes via FcγRIIa or FcγRIIIa [30], by ATP via P2X, P2Y, and adenosine receptors [31,32,33], and by complement-derived anaphylatoxins via the C3a and C5a receptors [34].

The number of mature MCs varies throughout the body, and a great number of mature MCs is present in the gastrointestinal (GI) tract, in which they are implicated in host defense. In particular, intestinal MCs are involved in the maintenance of homeostasis as well as in orchestrating local inflammation leading to the development of disease [35].

Based on the expression of different proteases stored in cytoplasmic granules, two main subsets of murine intestinal MCs have been identified [36]: MCs that are positive for mMCP-1 and mMCP-2 proteases are mainly located in the lamina propria close to the epithelium and for this reason are called mucosal MCs (MMCs); MCs that preferentially express in their granules the chymase mMCP-4, the elastase mMCP-5, and the tryptases mMCP-6 and 7 are found in the lower submucosa and are named connective tissue-type MCs (CTMCs). A third MC subtype, the interepithelial mucosal MCs (ieMMC), has been identified in mice [37]. The two mucosal subtypes, MMCs and ieMMCs, are rare in normal mouse GI, but increased during immune responses to intestinal helminth infections and in food allergies.

MC heterogeneity was also reported in the human GI tract, where two main subsets have been identified, mainly based on their differential expression of tryptase-β and chymase-1 [38]. Of note, 2 highly polymorphic genes (designated as hTPSAB1 and hTPSB2) give rise to different isoforms of tryptase-β [39], but their functional significance has not been evaluated experimentally. MCs that only express tryptase (MC_T_) are mainly present in the mucosal layer of the GI tract, while the dominant MC subset in the submucosa of the gut is characterized by the expression of tryptase, chymase, and carboxypeptidase A3 (CPA3) (MC_TCA_) [40,41]. A rare population of MCs exclusively expressing tryptase and CPA3, but not chymase, has recently been identified in the bronchial and esophageal mucosa of patients with asthma and eosinophilic esophagitis, respectively [42,43]. However, whether and how this subset contributes to human disease remains unknown.

These classifications are simplistic since they do not reflect the high level of intestinal MC plasticity due to the constant change in the local microenvironment. For instance, in respect to the small intestine, MCs that reside in the large intestine express higher amounts of toll-like receptors (TLRs) that are important for host defense against the abundant bacteria in the colon [44], demonstrating that regional diversity in microbiota composition can differently affect MC phenotype and function.

The advent of single-cell RNA sequencing (scRNAseq) technologies provides the great opportunity to analyze tissue-resident MCs, supporting the conclusion that MCs’ classification might be extended beyond the mucosal versus connective-like dichotomy across organs, including the gut [45,46,47].

## 3. Role of Intestinal MCs in Immune Homeostasis

Intestinal MCs play important homeostatic roles in the gut, controlling physiological processes such as the integrity and the baseline permeability of the gut’s epithelium mainly through the release of different granule proteases [35].

In humans, tryptase disturbs endocytic traffic and degradation of internalized antigens, allowing them to be easily transported across a damaged epithelial barrier [48].

A crucial role for the MC-released proteases in epithelial integrity was demonstrated by studies carried out on mMCP-4-null and mMCP-5-null B6 mice: these proteases mediate ischemia–reperfusion injury of skeletal muscle and thermal injury of skin mainly through the proteolytic disruption of tight junctions [49,50,51].

In the gut, the lack of mMCP-4 chymase increased crypt depth and decreased expression of the tight-junction protein claudin-3 on the lateral membranes of the epithelium with respect to a WT phenotype, revealing an important role for this chymase in the homeostatic regulation of intestinal epithelial anatomy and function [52].

### Crosstalk Between Microbiota and MCs in the Maintenance Gut Homeostasis

Emerging evidence supports the existence of a mutual crosstalk between intestinal MCs and the gut microbiota, which could have a strong impact on intestinal homeostasis [53,54].

Commensal bacteria promote the expression of CXCR2 ligands by intestinal epithelial cells, which, in turn, is responsible for MC migration into the intestine [55]. Accordingly, germ-free mice exhibit an impaired homing of gut MCs and do not develop food allergy upon oral sensitization [56].

Intestinal MCs respond to microbial antigens thanks to the expression on their membrane surface of TLR2 and TLR4 [57]. TLR2-mediated response consists of degranulation followed by cytokine release, while TLR4 activates the cytokine release without degranulation [58].

However, although some microorganisms can elicit an MC-driven pro-inflammatory response, other microorganisms are able to reduce MC activation, thus limiting inflammation and favoring homeostatic conditions.

For instance, in vitro coculture of MCs with some bacteria strains induces exocytosis of enzymes and proteases stored in MC granules and the release of pro-inflammatory cytokines and chemokines [59,60,61]. Moreover, human MC cultured with *Listeria monocytogenes* are able to reduce bacterial growth through the production of ROS and the consequent release of extracellular traps (MCETs) [62]. Similarly, upon *Candida albicans* recognition, human MCs were transiently able to release tryptase-containing MCETs to kill the opportunistic pathogen [63].

On the other hand, several commensal bacteria, such as *Enterococcus fecalis*, *Lactobacillus paracaseii*, and nonpathogenic *Escherichia coli*, utilize distinct inhibitory mechanisms to impair in vitro murine MC degranulation induced by IgE/antigen triggering [64,65]. Of note, in a model of murine atopic dermatitis, oral administration of *Enterococcus faecalis* reduced MC infiltration and serum IgE levels, ameliorating the pathology [66], supporting the ability of gut microbiota to limit in vivo MC functions.

Thus, it is likely that during dysbiosis, intestinal MCs rapidly respond to abnormal gut flora by releasing preformed and newly synthesized mediators, which contribute to promoting a local hyperinflammatory response [67].

Accordingly, supplementation with specific probiotic strains is considered a preventive/therapeutic strategy for dysbiosis management and immune homeostasis [68] and contributes to MC stabilization [69].

In recent years, several studies demonstrated that a microbial dysbiosis can be associated with many human inflammatory disorders [70,71].

## 4. Gut Microenvironment and MC Activation During Inflammation

MC ability to rapidly sense a changing environment and consequently adapt to the specific received triggers can explain the influence of the gut cytokines, growth factors, and microbial components in shaping the phenotype and functions of intestinal MCs observed in inflammatory disorders and during parasitic infections [70,71,72,73,74,75].

Following infection by *Trichinella spiralis* and *Trichuris muris*, the total number of MCs increases, and in the acute phase, a shift from a connective tissue-like phenotype to a mucosal phenotype is occurring with a consequent elevated expression of the protease MCP-1 [72,73,74]. MCP-1 appeared to be responsible for the degradation of occludin, thus increasing intestinal permeability and facilitating worm expulsion [73,74]. On the other hand, in the chronic phase of inflammation, Shin and co-authors underline a selective role of connective tissue-like MCs. Indeed, the tryptase MCP-6 was shown to be required for eosinophil recruitment and for the eradication of *Trichinella spiralis* [75].

Many studies have well established that MCs in the intestinal mucosa are the major effector cells in IgE-mediated food-induced disorders, including food allergies [76,77].

Several excellent articles summarize critical knowledge on the immune mechanisms of MC sensitization in detail [77,78,79,80]; thus, we touch only on some aspects responsible for the food induction of a T_H_2 cell-mediated inflammatory response in the gut.

Exposure to a certain food in the context of concomitant external and internal trigger(s) inducing tissue damage produces an alarmin signature [epithelium-derived interleukin-25 (IL-25), IL-33, and thymic stromal lymphopoietin (TSLP)] that converges on IL-12 inhibition on DCs and upregulation of Th2 polarizing co-stimulatory molecules [81].

Th2 cells activate food allergen-specific B cells, promoting the production of allergen-specific IgE.

Allergens induce cross-linking of IgE bound to FcεRI on the surface of MCs, promoting the release of pro-inflammatory mediators able to induce both local and systemic responses. In addition to controlling type I hypersensitivity reactions, in the gastrointestinal mucosa, MCs orchestrate the recruitment of tissue-infiltrating leukocytes that amplify type 2 tissue inflammation [77].

**Figure 1:**
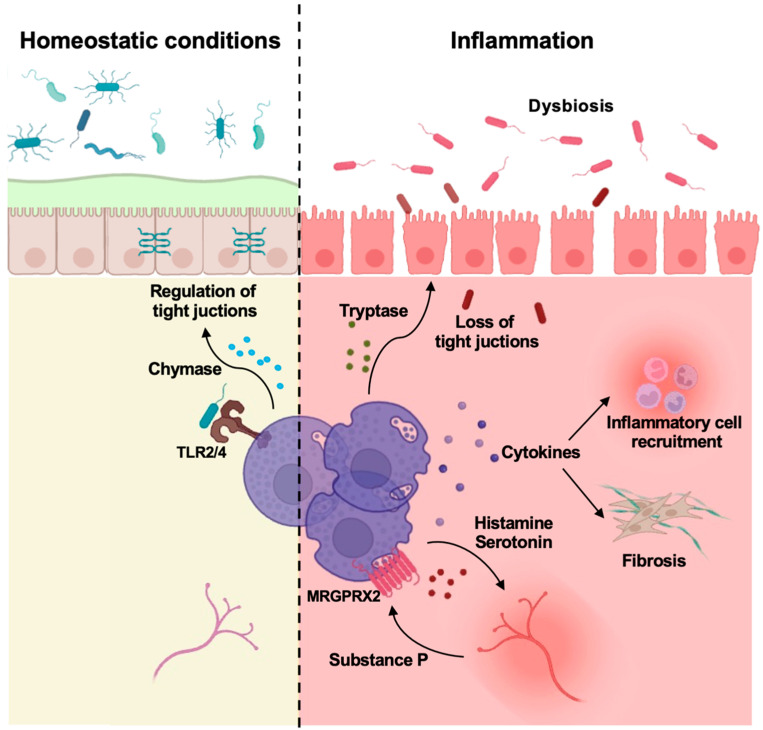
Mast cells control gut homeostasis and inflammation. Intestinal MCs are involved in the maintenance of homeostasis (**left side**) as well as in orchestrating local inflammation leading to the development of IBD (**right side**). The main effects of mast cell mediators are depicted. Modified from Van Remoortel S et al., Cell Mol Gastroenterol Hepatol. 2024, 18, 101391 [83] and created in https://BioRender.com.

Several studies have also demonstrated an increased number of activated MCs in the inflamed intestinal mucosa of patients affected by IBD and IBS, often associated with a concomitant stress response [6,82]. The implication of MCs and their selective mediators in disease progression is depicted in Figure 1 and will be discussed in the following paragraphs.

Of note, the presence of activated and degranulated MCs in the colon during the transition from gut inflammation to transformation supports MC implication in colon cancer (CRC) development [6]. However, the precise role of the different intestinal MC subsets in the diverse phases of tumor development is still a matter of debate [84,85].

Thus, intestinal MCs acquire a different behavior when faced with normal, damaged, or transformed epithelial cells in the gut, and eventually they can orchestrate deviated immune responses.

## 5. Characterization of Intestinal MCs in Patients with IBD

IBD, including ulcerative colitis (UC) and Crohn’s disease (CD), are complex multifactorial diseases of the gastrointestinal tract, triggered by environmental factors in genetically susceptible individuals [6]. Current therapies based on the use of monoclonal antibodies directed against cytokines offer amelioration and prolonged periods of remission but have important limitations. Indeed, during ulcerative colitis, more than 30% of patients do not initially respond to therapy, while others lose response over time [86]. Thus, new treatment strategies are needed.

Several studies have been performed to characterize the distribution and phenotype of MCs within the various intestinal segments of patients with IBD [7].

Initially, histological studies in humans performed by Bischoff and coauthors detected a reduced number of toluidine blue^+^ MCs in the involved intestinal segments of patients with IBD [87]. However, upon immunohistochemistry performed to evaluate the presence of hTryptase-β and hChymase-1, the same study discovered that a large portion of the immunoreactive proteases resided near but outside the MCs, suggesting that the apparent decreased number of MCs was due to MC degranulation.

Later, several authors provided convincing evidence of increased MC numbers in inflammatory bowel diseases, including UC and CD [88,89,90,91]. Nolte and coauthors found that MCs were increased in patients with UC compared with control subjects [88], while Nishida and colleagues found increased numbers of MCs in the upper part of lamina propria in patients with IBD [90]. A greater number of MCs was also observed in the hypertrophied and fibrotic tissue sites of CD patients compared with normal gut [89], suggesting a role for MCs in regulating intestinal fibrosis. Relevant to this, tryptases secreted from human MCs activate fibroblasts to differentiate into myofibroblasts, able to release more extracellular matrix (ECM) proteins during fibrosis changes revealed in IBD patients [92]. These findings have important clinical implications, providing support for the use of MC drugs to prevent IBD-induced intestinal fibrosis.

Not only the number of MCs was elevated, but also the expression levels of several MC mediators were greatly changed in IBD in comparison with normal subjects [82,86,87,88,89,90,91,92,93,94,95,96,97,98]. Early studies support MC contribution to mucosal inflammation demonstrating an increased level of MC-derived pro-inflammatory mediators in intestinal biopsies of IBD patients [93,94]. To further assess the degree of MC activation in patients with IBD, mucosa biopsies were placed in an oxygenation system for 4 h and the levels of tryptase-β and histamine were then measured in the supernatants: their exocytosis was more pronounced in inflamed tissue compared with noninflamed colon [95]. An increased secretion of tryptase and histamine has also been documented in biopsies from the duodenum, colon, and rectum of IBD patients [96,97]. These results are in line with a previous study revealing a positive correlation between the level of the histamine metabolite in the urine of IBD patients with the severity and the extent of disease [98].

Both histamine and tryptase can trigger nociceptive receptors leading to hypersensitivity of visceral sensory nerves [99]. Moreover, mouse and human tryptases can favor barrier dysfunctions, as revealed in acute experimental colitis and IBD [4,5,6].

Interestingly, many populations of degranulating MCs also release substantial amounts of TNF-α, IL-16, and substance P in the mucosa of the ileum and colon of patients with IBD [100,101], supporting a role for those MC-released mediators in the pathogenesis of IBD.

Regarding the specific MC subset(s) involved in IBD, a recent paper supported a pivotal role for a large subset of connective-like murine MCs expressing the Mas-related G protein-coupled receptor b2 (Mrgprb2) receptor in the development of colitis [83]. Mrgprb2-expressing MCs increased in the inflamed colon of WT-type mice and were found in proximity with nerve fibers, whereas Mrgprb2-/- mice showed a reduction in the influx of neutrophils and acute colitis progression [83].

Accordingly, by single-cell RNA technology, Chen and coauthors demonstrated the presence of a human MC subset expressing MRGPRX2 that plays a role in the development of UC through the establishment of a positive feedback inflammatory loop [102].

These results suggest the targeting of MRGPRX2 as a novel potential therapeutic strategy in UC. However, signals regulating the expansion/recruitment of this connective-like MC subset during colonic inflammation remain undefined.

Different IgE-independent mechanisms have been proposed as triggers for the activation of intestinal MCs in patients with IBD.

Stress factors can enhance mucosal MC degranulation, resulting in GI barrier impairment and intestinal inflammation [103,104,105]. In humans, the release of MC proteases into the lumen of the small intestine occurs in response to cold pain stress, while the release of tryptase and histamine, but not PGD2, is more pronounced in food-allergic patients than in healthy volunteers [106].

Ig-free light chains are also able to activate MCs and play a role in murine MC-dependent colitis and possibly also in human IBDs [107]. Although this finding is intriguing, the mechanism of MC activation remains largely unclear.

A high number of MCs that express both FcγRI and TLR4 has been reported in IBD patients [108], suggesting that in vivo a synergistic action of IgG and LPS may account for MC activation. Apart from TLR4, the intracellular bacterial receptor NOD2 was also found to be up-regulated in intestinal MCs of CD patients [109], suggesting its contribution in the recruitment and/or activation of MCs.

**Figure 2:**
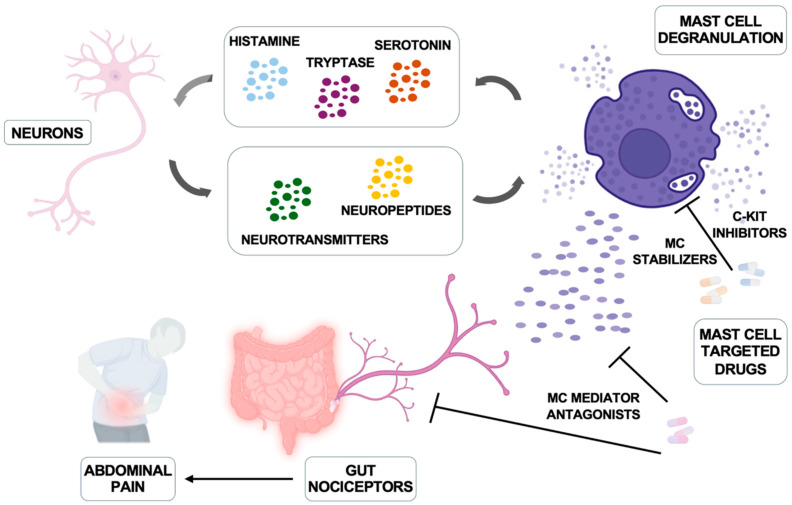
Mast cells and nerve bidirectional communication and therapeutic interventions. Neurons produce neuropeptides and hormones that trigger mast cell activation and degranulation; in turn, mast cells produce histamine, serotonin, and tryptase that can regulate neuronal function. The main inhibitors of mast cells and their mediators are illustrated. Modified from Jacobson A et al. Mucosal Immunology 2021, 14, 555–565 [111] and created in https://BioRender.com.

The amounts of neuropeptide(s) increased in IBD patients and their capability to support activation of both murine and human intestinal MCs has been reported [110], suggesting a possible role for MC/intestinal neuron cooperation in the pathogenesis of the disease (Figure 2).

## 6. Mast Cells and Irritable Bowel Syndrome

IBD and irritable bowel syndrome (IBS) are for the most part overlapping in terms of several symptoms, including abdominal pain and diarrhea. However, IBS is a functional gut disorder characterized by neuroinflammation and irregular digestive problems resulting from several non-pharmacological or pathological stimuli and by emotional feelings [112].

A first link between MCs and IBS was demonstrated by Weston and coauthors, reporting increased numbers of mucosal MCs in the terminal ileum of IBS patients [113]. Along the same line, Barbara and coauthors showed that patients suffering from IBS had more MCs in the left colon and rectum than those of normal individuals, and many of them resided near to nerve endings [114].

Once activated, those MCs release histamine and tryptase that may act on intestinal neurons through histamine receptors and proteinase-activated receptor (PAR-2) [115,116], thus explaining not only the pain but also gut sensorimotor dysfunction and related diarrhea in patients with IBS [117,118]. Thus, a bi-directional MC-nerve-interaction occurs, as illustrated in Figure 2: MCs trigger neurons for activation, and neuronal factors promote/amplify MC activation [6,111,119].

Interestingly, Aguillera-Lizarraga and coauthors described an IgE-dependent mechanism of MC activation caused by a brake in oral tolerance to dietary antigens induced by an inflammatory environment and resulted in food-induced abdominal pain [76]. However, the main MC subset implicated has not been characterized.

Upon MC activation, enhanced tryptase activity has been shown in ex vivo colonic biopsies obtained by patients with diarrhea-predominant IBS (IBS-D) [120]. On the contrary, patients with constipation-predominant IBS (IBS-C) show high levels of MC cysteine protease activity that positively correlates with disease severity and abdominal pain scoring [121].

Of note, upregulation of the gene encoding for the MRGPRX2 receptor has been observed in colonic biopsies of some IBS patients that also show high levels of IL-1b and prostaglandin synthase PTGS2 gene expression [122], supporting the presence in those patients of a connective-like MC subset that may contribute to the development of abdominal pain. However, to what extent this subgroup of patients could benefit from mast cell-targeted therapy remains to be investigated.

Thus, different environmental stimuli cause the onset of IBS-D or IBS-C symptoms, in which the involvement of specific MC-released mediators may be hypothesized. However, further investigations are needed to identify the main MC subset(s) involved.

## 7. Targeting MCs and Their Mediators in IBD and IBS

MCs and their derived mediators may represent targets for therapeutic options in patients with IBD and IBS.

The candidate drugs include MC stabilizers (ketotifen or cromoglycate), antagonists of MC mediators, and inhibitors of MC proteases [112], some of which show benefit to IBD and IBS patients (Table 1).

Treatment with the MC stabilizer ketotifen had shown beneficial effects in IBD and IBS patients [123,124,125,126]. Similarly, disodium cromoglycate (DSCG) administration resulted in a clinical improvement of symptoms in IBS-D patients by decreasing the release of tryptase but also in IBD [127,128,129].

Second-generation anti-histaminergic drugs, such as ebastine, prevent histamine-mediated signaling by blocking the histamine receptor H1 and represent the first-line treatment option for IgE-mediated MC disorders such as allergic rhinitis and chronic urticaria [130].

The effect of ebastine has been evaluated in a first pilot study on patients with IBS: up to 46% of patients receiving ebastine for 12 weeks reported significantly improved symptom relief and reduced abdominal pain, compared to 13% in the placebo-treated group [131]. More recently, a phase 2 randomized, placebo-controlled study further confirmed the potential of ebastine in improving global relief of hypersensitivity symptoms and abdominal pain intensity in IBS-D patients [132].

Blockade of serotonin may also represent another interesting approach to treat abdominal pain. Indeed, 5-HT3 antagonists, such as alosetron and ramosetron, have been repeatedly shown to be effective in IBS-D patients with reduction in abdominal pain and discomfort [133,134].

Serine protease inhibitors have proven efficacy as a treatment for visceral hypersensitivity in preclinical models of IBS [135,136]. Moreover, in a phase II study on UC patients, the tryptase inhibitor APC 2059 showed symptom improvement in more than 50% of the treated patients [137].

**Table 1:** Mast cell-targeted therapies in IBD and IBS.

**Targets**	**Drugs**	**Mechanism of Action**	**Clinical Outcomes**	**References**
**MC (activation and degranulation)**	Ketotifen Disodium Cromoglycate	Antagonize H_1_R and stabilize MCs Inhibit MC degranulation	Improve bowel functions of IBD (UC) and IBS-D patients Improve symptoms of IBD (UC) and IBS-D patients	[123,124,125,126] [127,128,129]
**MC mediators:** **Histamine** **Serotonin** **Tryptase** **Serine proteases**	Ebastin Ramosetron Alosetron APC2059	H_1_R antagonist 5-HT_3_ antagonists PAR2 antagonist	Improve visceral pain of IBS-D and IBS-NC patients Reduction of abdominal pain and discomfort in IBS-D and IBS-M patients Inhibit hypersensitivity and improve symptoms of IBD (UC) and IBS patients	[131,132] [133,134] [135,136,137]

H_1_R, histamine H_1_ receptor; 5-HT_3_, serotonin receptor subtype 3; IBD, irritable bowel disease; IBS, irritable bowel syndrome; IBS-D, diarrhea-predominant IBS; IBS-M, mixed IBS; IBS-NC, non-constipated IBS; MC, mast cells; PAR, protease-activated receptor; SCF, stem cell factor; UC, ulcerative colitis.

Considering the ability of specific bacterial or fungal strains to selectively modulate MC functions [69], the use of probiotics may also represent a therapeutic option to modulate MCs in patients with IBD and IBS. Indeed, the administration of probiotics displayed positive effects on the treatment of pouchitis [138], in the induction of remission in patients with UC [139,140], and in the reduction in symptoms and inflammation in patients suffering from IBS [141].

However, at present no recommendations regarding individual species, strains, or mixed compositions can be made because of the limited data available. Moreover, the mechanisms by which probiotics exert their effect are highly complex, and a better characterization of the main MC subset(s) involved is needed to identify more effective and safe therapies for patients.

Several studies have identified pro-inflammatory dietary patterns, such as those high in processed foods and low in fiber, as contributors to gastrointestinal symptoms in IBS [142,143]. Of note, injection of certain food antigens (gluten, wheat, soy, and milk) into the rectosigmoid mucosa of patients with IBS may promote MC activation [76].

However, further studies are required to reveal whether and how dietary components contribute to IBS to define the right diet for the prevention and/or the management of the gut syndrome.

## 8. Conclusions and Future Directions

MCs can carry out diverse functions in the gut. Indeed, they can exert a protective role that contributes to intestinal homeostasis, but they can also release potent pro-inflammatory mediators that exacerbate the many features of IBD and IBS. This shift in function is likely due to intestinal MC plasticity driven by cues from the altered tissue microenvironments. Furthermore, it is possible that a shift in the composition of the microbiota may affect the phenotype and the functions of MCs and may contribute to the increased prevalence of IBD in the last decade [70,71].

The release of pro-inflammatory mediators from the activated MCs in the gut of patients with IBD and IBS likely contributes to the inability of the epithelium to act as an effective barrier to pathogens.

The possibilities to suppress MC functions are quite limited since available drugs are restricted to MC stabilizers or antagonists of MC mediators. MC stabilizers have an effect on some IBD and IBS patients; however, the effects are rather weak and inconsistent. Although a reduction in abdominal pain and symptoms has been reported in IBS patients upon treatment with the histamine receptor H1 antagonist ebastine, histamine antagonists turned out to have limited effect in IBD patients, possibly because MC mediators other than histamine play a major role. Regarding the c-kit inhibitors, both Midostaurin and Avapritinib are only approved for the treatment of advanced systemic mastocytosis [144].

In conclusion, a better characterization of the main MC subset(s) involved in IBD and IBS is needed to identify more effective and safe therapies for patients.
